# Supplementary material for: Empirical methods for controlling false positives and estimating confidence in ChIP-Seq peaks
Source: BMC Bioinformatics. 2008 Dec 5;9:523. doi: 10.1186/1471-2105-9-523 (PMC2628906; doi:10.1186/1471-2105-9-523)
Supplement: Additional File 1 — A variety of html documents from the USeq web site detailing the available applications, their best usage, output file type descriptions, command line menus, etc. [file 1471-2105-9-523-S1.zip › USeqUserGuides/JavaDoc.zip!/JavaDoc/gata/main/GATAParams.html]

GATAParams


|  |  |  |
| --- | --- | --- |
| **Overview** \| **Package** \| **Class** \| **Use** \| **Tree** \| **Deprecated** \| **Index** \| **Help** | |  |
| **PREV CLASS**   **NEXT CLASS** | **FRAMES**    **NO FRAMES**     **All Classes** |  |
| SUMMARY: NESTED \| FIELD \| CONSTR \| METHOD | DETAIL: FIELD \| CONSTR \| METHOD |  |


---


## gata.main Class GATAParams

```
java.lang.Object
  gata.main.GATAParams
```

---

``` public class GATAParams extends java.lang.Object ```

**Author:**
:   Nix
    Master params file for the GATAPlotter program.

---

**Constructor Summary**

`GATAParams()`


| **Method Summary** | |
| --- | --- |
| `boolean` | `areGenericLabelsVis()` |
| `boolean` | `areLabelsVis()` |
| `double` | `getA()` |
| `AlignPanel` | `getAAlignPanel()` |
| `AlignPanel` | `getAlignPanel()` |
| `AlignParams` | `getAlignParams()` |
| `double` | `getB()` |
| `java.awt.Color` | `getBackgroundColor()` |
| `int` | `getBiggestAlignPanelY()` |
| `double` | `getC()` |
| `AnnoSpecParams` | `getCompAnnoSpecParams()` |
| `Console` | `getConsole()` |
| `double` | `getD()` |
| `java.awt.BasicStroke` | `getDashStroke()` |
| `float` | `getDefaultTrackThickness()` |
| `java.awt.Color` | `getDNAColor()` |
| `double` | `getE()` |
| `double` | `getF()` |
| `double` | `getFatLineThicknes()` |
| `java.awt.BasicStroke` | `getFatStroke()` |
| `java.awt.Font` | `getFont()` |
| `GATAFrame` | `getGataFrame()` |
| `GATAPlotter` | `getGataPlotter()` |
| `int` | `getHeight()` |
| `double` | `getHeightLabels()` |
| `java.awt.Color` | `getLabelColor()` |
| `java.awt.BasicStroke` | `getNormalStroke()` |
| `double` | `getNtPerPixel()` |
| `java.io.File` | `getObjectsFile()` |
| `double` | `getPixBtwDNATracks()` |
| `double` | `getPixBtwLabelTrack()` |
| `double` | `getPixBtwProtRNA()` |
| `double` | `getPixBtwTracks()` |
| `double` | `getPixBtwTransGrpLabel()` |
| `double` | `getPixBtwTransGrps()` |
| `double` | `getPixBtwTransGrpsGene()` |
| `float` | `getPixFatLineThickness()` |
| `double` | `getPixHeightArrows()` |
| `double` | `getPixNormalLineThickness()` |
| `double` | `getPixPerNt()` |
| `float` | `getPixThinLineThickness()` |
| `PlotterPreferences` | `getPlotterPreferences()` |
| `java.awt.Color` | `getProteinColor()` |
| `AnnoSpecParams` | `getRefAnnoSpecParams()` |
| `java.awt.Color` | `getRNAColor()` |
| `int` | `getSmallestAlignPanelY()` |
| `java.awt.BasicStroke` | `getThinStroke()` |
| `ToolsFrame` | `getToolsFrameRef()` |
| `int` | `getWidth()` |
| `int` | `getZoomedWidth()` |
| `boolean` | `isDNAVis()` |
| `boolean` | `isGenericLabelsVis()` |
| `boolean` | `isGffCompPresent()` |
| `boolean` | `isGffRefPresent()` |
| `boolean` | `isLabelsVis()` |
| `boolean` | `isProteinVis()` |
| `boolean` | `isRNAVis()` |
| `void` | `setA(double d)` |
| `void` | `setAAlignPanel(AlignPanel panel)` |
| `void` | `setAlignPanel(AlignPanel a)` |
| `void` | `setAlignParams(AlignParams ap)` |
| `void` | `setB(double d)` |
| `void` | `setBackgroundColor(java.awt.Color color)` |
| `void` | `setBiggestAlignPanelY(int i)` |
| `void` | `setC(double d)` |
| `void` | `setCompAnnoSpecParams(AnnoSpecParams params)` |
| `void` | `setConsole(Console console)` |
| `void` | `setD(double d)` |
| `void` | `setDefaultTrackThickness(float f)` |
| `void` | `setDNAColor(java.awt.Color color)` |
| `void` | `setDNAVis(boolean b)` |
| `void` | `setE(double d)` |
| `void` | `setF(double d)` |
| `void` | `setGataFrame(GATAFrame frame)` |
| `void` | `setGataPlotter(GATAPlotter gata)` |
| `void` | `setGenericLabelsVis(boolean b)` |
| `void` | `setGffCompPresent(boolean b)` |
| `void` | `setGffRefPresent(boolean b)` |
| `void` | `setHeight(int i)` |
| `void` | `setHeightLabels(double d)` |
| `void` | `setLabelColor(java.awt.Color color)` |
| `void` | `setLabelsVis(boolean b)` |
| `void` | `setNtPerPixel(double d)` |
| `void` | `setObjectsFile(java.io.File file)` |
| `void` | `setPixBtwDNATracks(double d)` |
| `void` | `setPixBtwLabelTrack(double d)` |
| `void` | `setPixBtwProtRNA(double d)` |
| `void` | `setPixBtwTracks(double d)` |
| `void` | `setPixBtwTransGrpLabel(double d)` |
| `void` | `setPixBtwTransGrps(double d)` |
| `void` | `setPixBtwTransGrpsGene(double d)` |
| `void` | `setPixFatLineThickness(float f)` |
| `void` | `setPixHeightArrows(double d)` |
| `void` | `setPixNormalLineThickness(float f)` |
| `void` | `setPixPerNt(double d)` |
| `void` | `setPixThinLineThickness(float f)` |
| `void` | `setPlotterPreferences(PlotterPreferences preferences)` |
| `void` | `setProteinColor(java.awt.Color color)` |
| `void` | `setProteinVis(boolean b)` |
| `void` | `setRefAnnoSpecParams(AnnoSpecParams params)` |
| `void` | `setRNAColor(java.awt.Color color)` |
| `void` | `setRNAVis(boolean b)` |
| `void` | `setSmallestAlignPanelY(int i)` |
| `void` | `setToolsFrameRef(ToolsFrame aTF)` |
| `void` | `setWidth(int i)` |
| `void` | `setZoomedWidth(int i)` |

**Methods inherited from class java.lang.Object**

`equals, getClass, hashCode, notify, notifyAll, toString, wait, wait, wait`

**Constructor Detail**

### GATAParams

```
public GATAParams()
```


**Method Detail**

### areGenericLabelsVis

```
public boolean areGenericLabelsVis()
```

---


### areLabelsVis

```
public boolean areLabelsVis()
```

---


### isDNAVis

```
public boolean isDNAVis()
```

---


### isGffRefPresent

```
public boolean isGffRefPresent()
```

---


### isGffCompPresent

```
public boolean isGffCompPresent()
```

---


### isProteinVis

```
public boolean isProteinVis()
```

---


### isRNAVis

```
public boolean isRNAVis()
```

---


### getA

```
public double getA()
```

---


### getAlignPanel

```
public AlignPanel getAlignPanel()
```

---


### getAlignParams

```
public AlignParams getAlignParams()
```

---


### getB

```
public double getB()
```

---


### getBackgroundColor

```
public java.awt.Color getBackgroundColor()
```

---


### getC

```
public double getC()
```

---


### getConsole

```
public Console getConsole()
```

---


### getD

```
public double getD()
```

---


### getDashStroke

```
public java.awt.BasicStroke getDashStroke()
```

---


### getDefaultTrackThickness

```
public float getDefaultTrackThickness()
```

---


### getDNAColor

```
public java.awt.Color getDNAColor()
```

---


### getE

```
public double getE()
```

---


### getF

```
public double getF()
```

---


### getFatLineThicknes

```
public double getFatLineThicknes()
```

---


### getFatStroke

```
public java.awt.BasicStroke getFatStroke()
```

---


### getFont

```
public java.awt.Font getFont()
```

---


### getHeight

```
public int getHeight()
```

---


### getHeightLabels

```
public double getHeightLabels()
```

---


### getLabelColor

```
public java.awt.Color getLabelColor()
```

---


### getNormalStroke

```
public java.awt.BasicStroke getNormalStroke()
```

---


### getNtPerPixel

```
public double getNtPerPixel()
```

---


### getPixBtwDNATracks

```
public double getPixBtwDNATracks()
```

---


### getPixBtwLabelTrack

```
public double getPixBtwLabelTrack()
```

---


### getPixBtwProtRNA

```
public double getPixBtwProtRNA()
```

---


### getPixBtwTracks

```
public double getPixBtwTracks()
```

---


### getPixBtwTransGrpLabel

```
public double getPixBtwTransGrpLabel()
```

---


### getPixBtwTransGrps

```
public double getPixBtwTransGrps()
```

---


### getPixBtwTransGrpsGene

```
public double getPixBtwTransGrpsGene()
```

---


### getPixFatLineThickness

```
public float getPixFatLineThickness()
```

---


### getPixHeightArrows

```
public double getPixHeightArrows()
```

---


### getPixNormalLineThickness

```
public double getPixNormalLineThickness()
```

---


### getPixPerNt

```
public double getPixPerNt()
```

---


### getProteinColor

```
public java.awt.Color getProteinColor()
```

---


### getRNAColor

```
public java.awt.Color getRNAColor()
```

---


### getThinStroke

```
public java.awt.BasicStroke getThinStroke()
```

---


### getToolsFrameRef

```
public ToolsFrame getToolsFrameRef()
```

---


### getWidth

```
public int getWidth()
```

---


### getZoomedWidth

```
public int getZoomedWidth()
```

---


### setAlignPanel

```
public void setAlignPanel(AlignPanel a)
```

---


### setAlignParams

```
public void setAlignParams(AlignParams ap)
```

---


### setConsole

```
public void setConsole(Console console)
```

---


### setHeightLabels

```
public void setHeightLabels(double d)
```

---


### setNtPerPixel

```
public void setNtPerPixel(double d)
```

---


### setPixBtwProtRNA

```
public void setPixBtwProtRNA(double d)
```

---


### setPixBtwTransGrps

```
public void setPixBtwTransGrps(double d)
```

---


### setPixBtwTransGrpsGene

```
public void setPixBtwTransGrpsGene(double d)
```

---


### setPixFatLineThickness

```
public void setPixFatLineThickness(float f)
```

---


### setPixHeightArrows

```
public void setPixHeightArrows(double d)
```

---


### setPixNormalLineThickness

```
public void setPixNormalLineThickness(float f)
```

---


### setPixPerNt

```
public void setPixPerNt(double d)
```

---


### setToolsFrameRef

```
public void setToolsFrameRef(ToolsFrame aTF)
```

---


### setZoomedWidth

```
public void setZoomedWidth(int i)
```

---


### getObjectsFile

```
public java.io.File getObjectsFile()
```

---


### setObjectsFile

```
public void setObjectsFile(java.io.File file)
```

---


### getGataPlotter

```
public GATAPlotter getGataPlotter()
```

---


### setGataPlotter

```
public void setGataPlotter(GATAPlotter gata)
```

---


### setGffRefPresent

```
public void setGffRefPresent(boolean b)
```

---


### setGffCompPresent

```
public void setGffCompPresent(boolean b)
```

---


### getAAlignPanel

```
public AlignPanel getAAlignPanel()
```

---


### isLabelsVis

```
public boolean isLabelsVis()
```

---


### getPixThinLineThickness

```
public float getPixThinLineThickness()
```

---


### setA

```
public void setA(double d)
```

---


### setAAlignPanel

```
public void setAAlignPanel(AlignPanel panel)
```

---


### setB

```
public void setB(double d)
```

---


### setBackgroundColor

```
public void setBackgroundColor(java.awt.Color color)
```

---


### setC

```
public void setC(double d)
```

---


### setD

```
public void setD(double d)
```

---


### setDNAColor

```
public void setDNAColor(java.awt.Color color)
```

---


### setDNAVis

```
public void setDNAVis(boolean b)
```

---


### setE

```
public void setE(double d)
```

---


### setF

```
public void setF(double d)
```

---


### setHeight

```
public void setHeight(int i)
```

---


### setLabelColor

```
public void setLabelColor(java.awt.Color color)
```

---


### setLabelsVis

```
public void setLabelsVis(boolean b)
```

---


### setPixBtwDNATracks

```
public void setPixBtwDNATracks(double d)
```

---


### setPixBtwLabelTrack

```
public void setPixBtwLabelTrack(double d)
```

---


### setPixBtwTracks

```
public void setPixBtwTracks(double d)
```

---


### setPixBtwTransGrpLabel

```
public void setPixBtwTransGrpLabel(double d)
```

---


### setPixThinLineThickness

```
public void setPixThinLineThickness(float f)
```

---


### setProteinColor

```
public void setProteinColor(java.awt.Color color)
```

---


### setProteinVis

```
public void setProteinVis(boolean b)
```

---


### setRNAColor

```
public void setRNAColor(java.awt.Color color)
```

---


### setRNAVis

```
public void setRNAVis(boolean b)
```

---


### setWidth

```
public void setWidth(int i)
```

---


### getGataFrame

```
public GATAFrame getGataFrame()
```

---


### setGataFrame

```
public void setGataFrame(GATAFrame frame)
```

---


### getBiggestAlignPanelY

```
public int getBiggestAlignPanelY()
```

---


### getSmallestAlignPanelY

```
public int getSmallestAlignPanelY()
```

---


### setBiggestAlignPanelY

```
public void setBiggestAlignPanelY(int i)
```

---


### setSmallestAlignPanelY

```
public void setSmallestAlignPanelY(int i)
```

---


### setDefaultTrackThickness

```
public void setDefaultTrackThickness(float f)
```

---


### isGenericLabelsVis

```
public boolean isGenericLabelsVis()
```

---


### setGenericLabelsVis

```
public void setGenericLabelsVis(boolean b)
```

---


### getPlotterPreferences

```
public PlotterPreferences getPlotterPreferences()
```

---


### setPlotterPreferences

```
public void setPlotterPreferences(PlotterPreferences preferences)
```

---


### getCompAnnoSpecParams

```
public AnnoSpecParams getCompAnnoSpecParams()
```

---


### getRefAnnoSpecParams

```
public AnnoSpecParams getRefAnnoSpecParams()
```

---


### setCompAnnoSpecParams

```
public void setCompAnnoSpecParams(AnnoSpecParams params)
```

---


### setRefAnnoSpecParams

```
public void setRefAnnoSpecParams(AnnoSpecParams params)
```


---


|  |  |  |
| --- | --- | --- |
| **Overview** \| **Package** \| **Class** \| **Use** \| **Tree** \| **Deprecated** \| **Index** \| **Help** | |  |
| **PREV CLASS**   **NEXT CLASS** | **FRAMES**    **NO FRAMES**     **All Classes** |  |
| SUMMARY: NESTED \| FIELD \| CONSTR \| METHOD | DETAIL: FIELD \| CONSTR \| METHOD |  |


---
